# What Training, Support, and Resourcing Do Health Professionals Need to Support People Using a Closed-Loop System? A Qualitative Interview Study with Health Professionals Involved in the Closed Loop from Onset in Type 1 Diabetes (CLOuD) Trial

**DOI:** 10.1089/dia.2019.0466

**Published:** 2020-05-29

**Authors:** Barbara Kimbell, David Rankin, Nicole L. Ashcroft, Lidiya Varghese, Janet M. Allen, Charlotte K. Boughton, Fiona Campbell, Atrayee Ghatak, Tabitha Randell, Rachel E.J. Besser, Nicola Trevelyan, Roman Hovorka, Julia Lawton

**Affiliations:** ^1^ Usher Institute, Medical School, University of Edinburgh, Edinburgh, United Kingdom.; ^2^ Wellcome Trust–Medical Research Institute of Metabolic Science, University of Cambridge, Cambridge, United Kingdom.; ^3^ Cambridge Clinical Trials Unit, Cambridge, United Kingdom.; ^4^ Department of Paediatrics, University of Cambridge, Cambridge, United Kingdom.; ^5^ Leeds Children's Hospital, Leeds, United Kingdom.; ^6^ Alder Hey Children's NHS Foundation Trust, Liverpool, United Kingdom.; ^7^ Nottingham Children's Hospital, Nottingham, United Kingdom.; ^8^ NIHR Oxford Biomedical Research Centre, John Radcliffe Hospital, Oxford University Hospitals NHS Foundation Trust, Oxford, United Kingdom; ^9^ Department of Paediatrics, University of Oxford, Oxford, United Kingdom.; ^10^ Southampton Children's Hospital, Southampton, United Kingdom.

**Keywords:** Closed-loop system, Health professional, Training, Qualitative Study

## Abstract

***Background:*** We explored health professionals' views about the training, support, and resourcing needed to support people using closed-loop technology in routine clinical care to help inform the development of formal guidance.

***Methods:*** Interviews were conducted with health professionals (*n* = 22) delivering the Closed Loop from Onset in Type 1 Diabetes (CLOuD) trial after they had ≥6 months' experience of supporting participants using a closed-loop system. Data were analyzed descriptively.

***Results:*** Interviewees described how, compared with other insulin regimens, teaching and supporting individuals to use a closed-loop system could be initially more time-consuming. However, they also noted that after an initial adjustment period, users had less need for initiating contact with the clinical team compared with people using pumps or multiple daily injections. Interviewees highlighted how a lessened need for ad hoc clinical input could result in new challenges; specifically, they had fewer opportunities to reinforce users' diabetes knowledge and skills and detect potential psychosocial problems. They also observed heightened anxiety among some parents due to the constant availability of data and unrealistic expectations about the system's capabilities. Interviewees noted that all local diabetes teams should be empowered to deliver closed-loop system care, but stressed that health professionals supporting closed-loop users in routine care will need comprehensive technology training and standardized clinical guidance.

***Conclusion:*** These findings constitute an important starting point for the development of formal guidance to support the rollout of closed-loop technology. Our recommendations, if actioned, will help limit the potential additional burden of introducing closed-loop systems in routine clinical care and help inform appropriate user education and support.

## Background

A closed-loop system is a rapidly evolving technology, which is predominantly used in management of type 1 diabetes, but is also being tested for use in type 2 diabetes. It combines a continuous glucose monitor (CGM), an insulin pump, and a control algorithm that interprets, in real time, CGM glucose data and calculates the amount of insulin needed to be administered by the pump. The first hybrid closed-loop system is now commercially available in the United States and Europe, and several more systems are expected to be launched within the year.^1^ The absence of available approved commercial systems has also prompted the use of do-it-yourself closed-loop systems, presenting health care professionals with additional ethical, regulatory, and practical challenges.^2^

To support rollout, it is important to learn from the perspectives and experiences of those who have already used, or supported the use of, closed-loop systems. To date, most studies have focused on the experiences of people with type 1 diabetes and/or their family members when using closed-loop systems.^3–13^ Health professionals' perspectives have only received limited attention, including their views about the training, support, and resourcing needed to support individuals using the technology in routine clinical care. This is an important omission, given the evidence that a key mediating factor in people's access to and experience of using diabetes technologies is professionals being appropriately trained and supported.^14^

To address this gap, we report findings from an interview study with health professionals involved in the Closed Loop from Onset in Type 1 Diabetes (CLOuD) study. CLOuD is an open-label, multicenter, randomized controlled trial, which is assessing the effect of closed-loop insulin delivery on residual beta-cell function in young people (aged 10–16 years) newly diagnosed with type 1 diabetes. In the first phase of the trial, participants were randomized to receive 24 months of treatment using either conventional multiple daily injection (MDI) therapy or closed-loop insulin delivery. Health professionals trained the adolescents and their families to use the closed-loop system and provided all study-related support. In most cases, the health professionals delivering the trial were also responsible for participants' routine clinical care (see Box 1 for further information about the trial, the study equipment, and the training and support provided to professionals delivering the trial and trial participants.) Key aims of the interview study were to (a) explore health professionals' experiences of providing training and support to individuals using the closed-loop system during the trial and lessons learnt for supporting future users and (b) seek their views about the training and resourcing health professionals will need to support people using closed-loop systems in routine clinical care to help inform development of formal guidance.

**Box 1. Details About the CLOuD Trial, Study Device, and Training Provided**

The Closed Loop from Onset in Type 1 Diabetes (CLOuD) trial aims to determine whether continued intensive metabolic control using a hybrid closed-loop system is better able to preserve beta-cell function in young people newly diagnosed with type 1 diabetes than standard multiple daily injection (MDI) therapy. The automated hybrid closed-loop system used in the first phase of the CLOuD trial, FlorenceM, comprised the following:

- A modified Medtronic 640G pump
- A Medtronic Guardian 3 sensor
- A locked-down Android smartphone with Medtronic enclosure containing the Cambridge model predictive control algorithm enabling wireless communication with the insulin pump

Eligibility criteria for the trial included the following: a diagnosis of type 1 diabetes within the preceding 21 days; aged between 10 and 16.9 years; a willingness to perform regular capillary blood glucose monitoring (at least four blood glucose measurements every day); to wear the study devices; and to upload pump and sensor data at regular intervals.

**Training and support provided to trial participants**

Prerandomization, all participants and their families received structured diabetes education and training in accordance with standard clinical practice as well as training on the MDI regimen. Participants randomized to the closed-loop arm were typically set up on the system over three visits to the clinic or research facility involving insulin pump training and initiation (3–4 h), continuous glucose monitor (CGM) training and initiation (2 h), and closed-loop initiation (3–4 h). Initiation on the closed-loop system also covered discontinuation of the closed-loop system, switching between closed-loop and standard insulin pump therapy, meal bolus procedure, and using the study devices during exercise. A closed-loop system user manual, including a trouble-shooting section, was also provided.

The study involved 14 planned visits and 1 telephone/e-mail contact in each arm over the 24-month study period. Participants were contacted by e-mail/telephone within one week after study initiation and subsequently followed up at three-monthly study visits to record any adverse events, device deficiencies, and changes to insulin doses; take an HbA1c sample; download data from the study devices; and fit participants of both study arms with a blinded CGM sensor to be worn for the next 14 days. Participants also had access to a 24-h telephone helpline to contact their local study team with any study-related matters. In most sites, the health professionals delivering the trial were also responsible for participants' routine clinical care during the trial.

**Training and support provided to site teams delivering the trial**

The trial was conducted in seven U.K.-based NHS sites with pediatric diabetes clinics: Addenbrooke's Hospital, Cambridge; Leeds Children's Hospital, Leeds; Alder Hey Children's Hospital, Liverpool; Nottingham Children's Hospital, Nottingham; Oxford Children's Hospital, Oxford; Southampton Children's Hospital, Southampton; and Royal Hospital for Children and Young People, Edinburgh. All sites were experienced in the use of insulin pumps and CGM devices, but their broader experience with closed-loop systems was limited at the time when the research was conducted.

Each participating center received a training visit from two members of the research team, who demonstrated the different study devices (pump, sensor, and study handset) and set up a working closed-loop system in real time. They also explained data downloads and the practicalities of the study, including details of the participant visits. All site staff delivering the trial completed a competency checklist. The research team also supported the centers on a second visit when they started their first participant on the closed-loop technology. Site teams received a trouble-shooting guide and had access to 24-h telephone support from the research team throughout the study.

## Methods

Qualitative methods facilitate the exploration of poorly understood topics as they allow findings to emerge from the data rather than from a priori hypotheses.^15^ We used semistructured interviews informed by topic guides to help ensure that the discussion remained relevant to the study aims while allowing participants to raise issues they considered important. Data collection and analysis took place concurrently to allow (unanticipated) findings from early interviews to be explored in later ones.

Our epistemological position was informed by the literature on the evaluation of complex health interventions^16^ as well as earlier work, which has highlighted how unexpected issues, benefits, and challenges may arise from introducing and using new diabetes technologies.^12,13^

### Recruitment

We recruited health professionals (doctors, diabetes nurses, and research nurses) in all seven participating trial sites (see Box 1 for details). Health professionals were invited to opt in to the interview study after they had at least 6 months' experience of supporting people using closed-loop systems during the trial. Recruitment continued until there was good representation of staff involved in trial delivery from across all sites (specifically, we sought representation from at least one doctor, one diabetes nurse, and one research nurse from each site) and data saturation was reached (i.e., no new findings were identified in new data collected).

### Data collection and analysis

B.K., an experienced, non-clinical qualitative researcher, conducted the interviews using a topic guide informed by literature reviews and inputs from clinical coinvestigators and revised in response to emerging findings. Key topic areas relevant to the reporting in this article are outlined in Box 2. The interviews took place between August 2018 and June 2019, averaged 70 min, and were digitally recorded and transcribed in full.

**Box 2. Key Topic Areas Explored in the Interviews**

- Interviewees' clinical background, training, and experience; previous involvement (if any) in trials of closed-loop technology.
- Experiences of training study participants to use the closed-loop system; perceived differences to training people using conventional insulin regimens.
- Experiences of providing support to participants using a closed-loop system; perceived differences in the type and amount of support required compared with people using other approaches; perceived sustainability of this level of support upon rollout.
- Experiences of the training and support received to deliver the trial; views about what kind of training, support, and resources health professionals will need to support closed-loop users in routine clinical care.
- Views about who should deliver closed-loop routine care.
- Perceived impact of the rollout of closed-loop technology on workloads and wider health care resources.

We undertook qualitative, descriptive data analysis, which produces low-inference descriptions of views and experiences and is particularly suited to understanding and illuminating issues relevant to policy and practice.^17,18^ B.K. and J.L. undertook independent analyses, which involved repeatedly reading and cross-comparing individual transcripts and writing separate reports, before meeting to discuss their interpretations of the data and agreeing on a coding frame that captured key areas of relevance to clinical practice development. The use of a qualitative analysis software package, NVivo10 (QSR International, Doncaster, Australia), facilitated data coding and retrieval. Coded datasets were subjected to further analysis to develop more nuanced interpretations of the data and identify representative quotations. Informed by a “What? So what? Now what?” approach to facilitating collaborative working between practitioners and researchers,^19^ study findings were presented to members of the wider CLOuD trial consortium to generate realistic and practical recommendations appropriate for a range of diabetes professionals and clinical settings.

The Usher Research Ethics Group (UREG), University of Edinburgh, granted ethical approval for the study (approval date: February 8, 2018). To safeguard anonymity, we use unique identifiers in the reporting below (i.e., D = doctor; N = diabetes nurse; and RN = research nurse).

## Results

The sample comprised 22 health professionals (7 doctors, 9 diabetes nurses, and 6 research nurses). Further details are provided in Table 1.

**Table 1.** Participant Characteristics

	N	%
CLOuD sites (*n* = 7)		
Total number of interviewees	22	
Interviewees per site—range (mode)	1–5 (4)	
Role		
Diabetes consultants	7	32
Diabetes nurses	9	41
Research nurses	6	27
Years of diabetes experience		
<5 years	6	27.3
5–10 years	5	22.7
>10 years	11	50
Interviewees with previous closed-loop trial experience	5	23

CLOuD, Closed Loop from Onset in Type 1 Diabetes.

Below, we report interviewees' experiences of training and supporting young people and their families to use the closed-loop system; their views about what training and resourcing diabetes professionals will need to support people using the system in routine clinical care; and their views about who would be best placed to deliver this support when the technology becomes more widely available. As responses were not found to differ according to health professionals' roles or previous experience with closed-loop system trials, individual characteristics are not separated out in the reporting below.

### Teaching and supporting people to use the closed-loop system

Interviewees reported that they had found teaching families how to use the closed-loop system to be straightforward (see Box 1 for information about the training provided to closed-loop participants). Some attributed this to individuals' growing familiarity and competency with technologies in everyday life:

“They all seem to take to it really well. I don't know whether it's now with younger ones being into technology more than I would have been. You know, I would have maybe struggled, but even the parents have all commented about how quickly they've picked up the use of the closed loop.” (RN1)

However, all interviewees noted that participants had needed more extensive teaching input than was typically required by individuals commencing use of MDI or continuous subcutaneous insulin infusion (CSII) therapy. Some reflected on the fact that trial participants were newly diagnosed and so only had a rudimentary understanding of diabetes management, which could require the inclusion of more general diabetes education alongside their closed-loop system training. Interviewees also observed how providing education on the closed-loop system's multiple constituent parts (i.e., insulin pump, sensor, and handset interface) required more time than single-device training. Many interviewees described experimenting with different educational delivery approaches: “I tried to do as much as possible within just one visit and failed epically, so after that I've been a lot more structured and have split things up” (N5). All interviewees concluded that delivering closed-loop education had worked best when the system's component parts were introduced one by one at a pace appropriate to the individual, typically over several weeks, “so they have time to get used to one thing before they get the next” (N3).

Interviewees further observed that occasional technical glitches with the prototype equipment, and adolescents and parents needing to become accustomed to the individual devices and how to use these effectively, resulted in them initially needing more support than individuals using other insulin regimens. However, several described how these technical problems and early confidence issues were quickly overcome and how many users had subsequently sought less clinical input than was typically requested by people using other treatment approaches. To account for this, these interviewees noted that because the closed-loop system automatically managed any excessive variability in blood glucose levels, “there's very little, you know, background basal rates, compliance, insulin-to-carb ratio correction, target glucose, exercise, there's not that much that I need to do” (D7). As a result, interviewees noted how the initial increased effort required to set up newly diagnosed individuals on a closed-loop system was offset by reduced requirements for clinical intervention thereafter:

“There's so much equipment to get used to…So at the start, definitely they need more support, no question…I think after the support is…if they're using the closed loop well, support in terms of adjusting insulin is virtually nil.” (D2)

### Unanticipated consequences; implications for workload

Some interviewees observed that this reduced contact could have unanticipated consequences. They explained that when individuals started CSII therapy, they would normally use the initial weeks and months of enhanced contact to proactively educate users how to interpret patterns in their (or their child's) blood glucose data and determine appropriate adjustments to basal rates and mealtime ratios. Closed-loop users' reduced need for this type of support thus meant that there were fewer teaching opportunities, which could result in individuals “missing out really on the sort of basic education of what to do if things weren't going as well” (N9).

Interviewees also observed how reduced contact could affect more holistic aspects of diabetes care and potentially result in support needs emerging further down the line. N7, for example, remarked that diabetes professionals might “drop the ball in other areas, you know, psychosocial stuff that we'd pick up if we were speaking to them more regularly” (N7). Several interviewees also suggested that the system's efficiency in controlling glycemic excursions could delay some individuals coming to terms with their diagnosis by masking the true burden of having diabetes. Similarly, some noted that the system's ability to counteract neglectful self-management practices, such as lax carbohydrate counting or missed mealtime boluses, could mask underlying issues, such as a poor emotional adjustment to diabetes, which was consequently “probably being picked up later than it could have been” (D3).

Several interviewees observed that the closed-loop system could heighten some parents' anxieties due to the greater availability of real-time data. They described how having access to this information compelled some anxious parents to micromanage by overriding some of the closed-loop's automated adjustments and administering their own correction doses, thereby interfering with the system's ability to adapt to the user's individual insulin requirements. Some worried parents were also reported to have made frequent contact with clinicians to discuss what they erroneously perceived as dangerous blood glucose digressions, because “they don't have any experience of what usual type 1 diabetes glucose levels are like” (D5). As well as offering education to parents to address these kinds of misconceptions, interviewees highlighted the importance of proactively managing their expectations of the closed-loop system:

“I suppose you do have to make it clear that…it will do its best, but you are diabetic. You will have highs still and you will have lows still. Because I think they think that this will delete them full stop.” (RN5)

### Training and resources needed to support closed-loop users in routine care

Interviewees generally considered themselves proficient with the pump and sensor technology, so their main training needs to support people using a closed-loop system cohered around the handset containing the system's algorithm, which they found “reasonably straightforward, it's not difficult to learn” (N6). Interviewees felt that, while it was not necessary for everyone to be trained to educator level, all team members should have at least some understanding of the system: “I think all the clinicians…should be aware and understand the functionalities and know a little bit of troubleshooting” (D4). They also offered suggestions for how future health professional training might be delivered. This included training videos, Webinars, recorded Technology, Entertainment, Design (TED) talks, and in-depth training from device manufacturers “to provide specific competencies that we have proven to the company trainers that we are able to follow” (N5). Similarly, several interviewees recommended a competency assessment on completion of training, while others highlighted the need for a formal, accredited training scheme. Regardless of the mode of training delivery, all interviewees emphasized the importance of being given opportunities to familiarize themselves with the technology before use in clinical practice:

“I think webinars have their place…and recorded training. But you'd need to hold the kit…to actually have the kit in your hands and to manipulate it yourself…We wouldn't expect that the patients, you know, learn about how to do an injection from watching a video.” (N2)

Several interviewees also described how they would routinely test new insulin pumps and sensors on themselves to further their own understanding or demonstrate their use to families and how this had not been logistically feasible with the closed-loop system. Consequently, they suggested that upon wider rollout, health professionals should have access to simulation equipment to aid their own learning as well as a demonstration system to support education sessions with families. Interviewees also noted that having a manual available for ongoing reference was helpful, because “sometimes it's quite a gap between…someone going on closed-loop and when your next one goes onto it…so you need to go back and refer to it” (N8).

Interviewees also suggested that diabetes professionals should be issued with guidance on how to advise closed-loop users clinically. This included advice about more atypical cases, such as how best to support a very active person, and non-routine events such as travel across time zones. Additionally, D3, like others, suggested that guidance should highlight the most clinically important areas within the system's extensive data outputs, because “there are I think at least like 40, 50 pages of download that happen and…we've got to go through all of it, or the most pertinent aspects of it, in a flash, so knowing what bits will be useful and why in a clinic setting would be helpful” (D3). Relatedly, all interviewees emphasized that centers will require robust IT systems with good internet connectivity to facilitate these large data downloads.

### Who should provide closed-loop care?

All interviewees stressed that to be able to appropriately advise and support closed-loop users, health professionals needed to be proficient in the use of insulin pumps and sensors:

“I think the prerequisite should be complete familiarity with pumps and CGM…because it's a closed-loop and it will do pretty much all the work itself, it cannot be delivered by people who are not completely conversant with the parts of the loop.” (D2)

Some, however, noted that this expertise was currently lacking in some diabetes centers and hence the closed-loop system care should, at least initially, be managed by specialized centers of excellence:

“I've worked in quite a few different diabetes centres and have often been the only person with any pump experience there…I think getting everybody comfortable with delivering training for patients on closed-loop is probably a bit of a step too far, so at least starting with specialised centres is probably the most economical way of delivering it in the future.” (D5)

Conversely, many interviewees agreed with N6's sentiment that “there's no reason why every specialist diabetes team in every center can't take on that role. It's not any more rocket science than what people are managing with the pumps…and the CGM that's routine now” (N6). Indeed, several argued that it was in users' best clinical interests that local diabetes centers have knowledge of closed-loop technology to ensure their appropriate support in times of emergency, because “unfortunately lots of children do have emergencies with their diabetes…unless the local service is able to understand their insulin regimen, it is dangerous” (D6).

## Discussion

We report on health professionals' perspectives regarding the issues, opportunities, and challenges that may arise from the introduction and mainstreaming of closed-loop technology in routine clinical care. Our findings provide an important starting point for the development of formal guidance to support the rollout of closed-loop technology in routine clinical care, particularly with regard to health professionals' training and resource requirements. Below, we discuss and put forward our recommendations for potential next steps (see Box 3 for a summary of these recommendations).

**Box 3. Summary of Recommendations**

- Each service delivery hub should have at least one health professional with formal training in closed-loop technology.
- Health professionals wishing to provide closed-loop system care should undergo accredited training and evidence relevant continued professional development.
- Develop formal standards, which set out the core competencies expected of health professionals delivering closed-loop system education and care.
- Closed-loop system training for professionals should accommodate different modes of learning, include competency assessments and accredited options endorsed by relevant organizations, and provide guidance on available systems and their suitability for different types of users.
- Health professionals should have access and be encouraged to use demonstration closed-loop systems to understand their functionality and modes of use.
- Develop clinical guidance to support closed-loop system consultations.
- Develop a structured, stepwise education package for closed-loop system users.
- Ensure regular psychological assessment of closed-loop system users in line with existing clinical practice for supporting people with type 1 diabetes. Consider the use of additional validated psychological tools alongside initial user education.

Interviewees emphasized that the health professionals training and supporting people to use a closed-loop system must be proficient with current pump and CGM technologies. However, some indicated that not all diabetes teams currently have this level of competency. In studies of other diabetes technologies, similar shortfalls in expertise have been linked to time constraints, health professionals having limited device exposure, and a lack of consensus or policy regarding training requirements.^14,20^ In light of potential shortages in the requisite expertise, some interviewees in the current study suggested that, at least initially, only specialist centers should deliver closed-loop system care. Conversely, others highlighted the importance of local diabetes teams having the necessary technological know-how as they are typically the first port of call for diabetes-related emergencies. To address these issues, we propose that each service delivery hub should have at least one health professional with formal training in closed-loop technology to act as an expert resource for their local diabetes team. Additionally, as suboptimal user education and support can compromise both user safety and the system's clinical benefits and cost-effectiveness, any health professional wishing to provide closed-loop system care should undergo accredited training and evidence relevant continued professional development. Finally, the development of formal standards, which set out the core competencies expected of health professionals delivering closed-loop system education and care, should be considered.

Interviewees expressed preferences for a range of modes of learning, which included competency assessments, accredited training routes, and a need for experiential learning. Considered an ethical imperative by some,^21^ simulation-based learning in medical education is recognized as enhancing health professionals' knowledge and skills (and thus patient safety) as well as benefitting wider organizational outcomes, such as staff retention and positive culture change.^22^ Interviewees also suggested that health professionals should be provided with guidance to support closed-loop system consultations in routine clinical care. In response to these findings, we propose that closed-loop system training for professionals should be made available using different media, such as YouTube, TED talks and written materials, to accommodate different learning styles and flexible access. This should include competency assessments and formal accredited options, which (for quality assurance) have been endorsed by relevant learned organizations. Given the range of commercial closed-loop systems about to enter the market,^1^ training for health professionals should also include up-to-date guidance on available models and their suitability for different types of users. To facilitate experiential learning, we recommend that device manufacturers provide demonstration systems to help health professionals understand closed-loop system functionality and modes of use. Finally, we suggest that clinical guidance should be developed to support professionals delivering closed-loop system consultations. As interviewees highlighted, this resource should provide advice on basic troubleshooting; how to advise users clinically, including the consideration of atypical cases and nonroutine events; and which data fields to prioritize during time-pressured clinic appointments. This guidance could also be tailored to the specific needs of different age groups such as young children, adolescents, and adult users.

Interviewees described how delivering closed-loop system education had worked best when using a structured approach that introduced each component in turn. However, it must be noted that the CLOuD trial involved newly-diagnosed individuals; hence, users with prior experience of pumps and/or sensors are likely to have fewer training needs. Interviewees further observed that once set up on the system, its ability to reduce glycemic excursions lessened users' need for ad hoc clinical input compared with people using CSII and MDI. This prompted concerns about having fewer opportunities to reinforce critical diabetes management skills, such as how to independently adjust basal rates and mealtime ratios in the event of device failure. Resonating with others' observations, interviewees also described how some people had held unrealistic expectations about the system's capabilities^9,12^ and could feel overly anxious due to the greater availability of real-time blood glucose data.^23^ To address these concerns, we recommend the development of a structured, stepwise education package. This resource should instruct users on how to safely manage blood glucose in the event of device failure, set realistic expectations about time in range and glycemic excursions when using a closed-loop system, and to help minimize potential data burden, outline which aspects of the available data users should focus on.

Interviewees also noted how reduced contact with users, alongside the system's ability to rectify (and therefore mask) poor self-management practices (e.g., meal boluses being miscalculated or omitted), might compromise timely detection of psychosocial problems. Previous studies^24,25^ have shown how the closed-loop system can partially compensate for lax dietary management practices commonly observed among adolescents.^26,27^ To date, however, none have reported a potential for unintended psychological consequences to arise from the system's ability to make these compensations. Others have highlighted how diabetes-related depressive symptoms may lead to adolescents discontinuing the use of diabetes technologies.^28^ Thus, we recommend that health professionals regularly assess users of closed-loop systems, particularly adolescents, in line with existing clinical practice for the psychological support of people with type 1 diabetes. Furthermore, integrating validated psychological tools such as INSPIRE^29^ into users' initial teaching package could help clarify their expectations, anxieties, and hopes regarding closed-loop systems and highlight issues that may need to be addressed to ensure safe and effective long-term use of this technology.

A key study strength is the use of a flexible open-ended approach, which enabled us to identify issues and challenges previously unreported in the literature. A potential limitation is that all centers running the trial were experienced in the use of insulin pumps and CGMs, which may not be representative of other sites delivering diabetes care. Furthermore, studies have shown that health professionals may hold erroneous views regarding individuals' capabilities and suitability for diabetes technologies.^14,30^ Such preconceptions, as well as their experience of working on a clinical trial, may have biased the professionals in this study; indeed, we described how these professionals acknowledge such prejudices, and the trial's influence on their views, in a companion article.^31^ Similarly, their perspectives may reflect a more traditional paternalistic stance toward health care delivery and a difficulty accepting increased patient autonomy.^32^ We also focused on the experiences of health professionals working with a specific participant group (newly-diagnosed adolescents) and using one type of prototype closed-loop system. Future research could explore the perspectives of health professionals working in adult diabetes care or with different types of closed-loop technologies. It should also be noted that in the event of the CLOuD trial demonstrating clear clinical benefits to people using closed-loop technology from diagnosis, the number of newly-diagnosed individuals being moved onto closed-loop systems will likely increase, thereby strengthening the generalizability of our findings. Consequently, future work should consider longer-term follow-up of newly diagnosed closed-loop system users to establish whether health professionals' concerns regarding a delayed emergence of support needs are realized.

The above recommendations constitute an important and timely starting point for informing the mainstreaming of closed-loop systems in routine clinical care. If actioned by the relevant stakeholders, such as industry, health care providers, and pertinent learned societies, they will help limit the potential additional burden of introducing closed-loop systems in routine clinical care and help inform appropriate user education and support.

## Notes

^*^ Cambridge Clinical Trials Unit, Nicole Ashcroft, Josephine Hayes and Matthew Haydock (Institute of Metabolic Science, University of Cambridge) provided administrative support, NIHR Cambridge Clinical Research Facility supported the research team in their research-related activities, Artificial pancreas focus group contributors provided feedback on the study design.
